# Gender policies and advertising and marketing practices that affect women's health

**DOI:** 10.3402/gha.v6i0.20372

**Published:** 2013-06-26

**Authors:** Belén Cambronero-Saiz

**Affiliations:** Department of Public Health, University of Alicante, Alicante, Spain

**Keywords:** abortion, public policy, work-family reconciliation, pharmaceutical advertising, gender awareness communication campaigns, gender bias

## Abstract

**Background:**

The three papers of this doctoral thesis are based on the social construction of reality through the analysis of communication relating to health issues. We have analysed the contents of parliamentary, institutional, and mass media to uncover whether their communications create, transmit, and perpetuate gender biases and/or stereotypes, which may have an impact on people's health, with a particular focus on women.

**Objective:**

To analyse decision making and the creation of gender awareness policies and actions affecting women's health: (1) political debates about abortion, (2) gender awareness communication campaigns and educational actions, and (3) pharmaceutical advertising strategies.

**Design:**

Quantitative and qualitative methods were employed, and the research included observational studies and systematic reviews. To apply a gender perspective, we used the level of gender observation proposed by S. Harding, which states that: (1) gender is the basis of social norms and (2) gender is one of the organisers of the social structure.

**Results:**

Sixty percentage of the bills concerning abortion introduced in the Spanish Parliament were initiated and led by pro-choice women's groups. Seventy-nine percent of institutional initiatives aimed at promoting equality awareness and were in the form of educational actions, while unconventional advertising accounted for 6 percent. Both initiatives focused on occupational equality, and very few actions addressed issues such as shared responsibility or public policy. With regard to pharmaceutical advertising, similar traditional male–female gender roles were used between 1975 and 2005.

**Conclusions:**

Gender sensitivity continues to be essential in changing the established gender system in Spanish institutions, which has a direct and indirect impact on health. Greater participation of women in public policy and decision-making are critical for womens’ health, such as the issue of abortion. The predominance of women as the target group of institutional gender awareness campaigns proves that the gender perspective still lacks the promotion of shared responsibilities between men and women. There is a need for institutions that act as ‘policy watchdogs’ to control the gender biases in mass media and pharmaceutical marketing as well as to ensure the proper implementation and maintenance of Spanish equality laws.

Integrating a gender perspective in health implies taking into account relational factors when addressing health problems (1, 2), for example, by exploring differences in the socialisation between men and women with regard to family roles, job prospects, and types of occupation in order to understand patterns of health and disease (3–7).

The WHO Commission on Social Determinants of Health has warned that health differences become health inequalities when they stem from unfair and avoidable situations (8–10). Consequently, gender becomes an indicator of health inequality when gender roles that involve different levels of exposure to risk are accepted (8). Assigning the role of family caregiver exclusively to women has resulted in the feminisation of part-time work (11–14) and the horizontal segregation (masculinisation of professions) and vertical segregation (the glass ceiling) of paid work (15). Thus, women are exposed to different health risks, because they have a lower income (material risk) (16), a heavier workload (physical risk) (17–22), and less bargaining power, both on a personal and professional level (behavioural risk) (23).

## Gender perspective in the social construction of reality

The three papers are based on the social construction of reality. They analyse the contents of parliamentary, institutional, and mass media to uncover whether their communications create, transmit, and perpetuate gender biases and/or stereotypes, which may have an impact on people's health, with a particular focus on women.

R. Braidotti considers gender as ‘the multiple and complex ways in which social differences between the sexes acquire meaning and become structural factors in the organisation of social life’ (24). This reflects the idea of gender as a social construct that depends on ideological, cultural, religious, economic, ethnic, and historical factors.

P. Luckmann and T. Berger were the first to theorise that reality is socially constructed through the transformation of a social circumstance into an objective reality (25). In complex societies, gender roles have been standardised and institutionalised, and reflect society's views about what it means to be a woman or a man and how they are expected to interact with each another. This set of concepts forms the symbolic universe (25, 26). According to social constructionism, the assignment of gender roles is based on patriarchal social structures legitimised by repetition, which still persists in Spanish society today.

In this context, S. Harding provides an analytical framework consistent with the social construction of reality theory (27). She states that gender identity can be changed by social interaction, leading to the creation of male and female patterns of behaviour associated with sex, or by the role of gender as the primary organising force behind the society structure, whereby male and female roles in that society are assigned based on sex (27–29).

This thesis also employs the feminist communication theory (30), which states that communication can be used as a tool for changing or reinforcing the socially constructed symbolic universe. It also notes the importance of equal participation in power structures, as this ensures that the specific needs of women are taken into account (30–34).

## Aim and methodology

Based on the theories summarised above, the aim of this study is to analyse decision-making and the creation of gender awareness policies and actions affecting women's health (political debates about abortion, gender awareness communication campaigns and educational actions) and pharmaceutical advertising and marketing strategies (Table 1).

**Table 1:** Thesis summary

Theoretical justification	Explanatory theories of health inequities Theory of the social construction of reality from a gender perspective Feminist communication theory		
Aim	To analyse decision-making and the creation of gender awareness policies and actions affecting women's health (political debates about abortion, and gender awareness communication campaigns and educational actions) and pharmaceutical advertising and marketing strategies.		
Observation context: gender/health	Article I: Abortion	Article II: Work–life balance	Article III: Pharmaceutical advertising
Analysis of secondary data	Parliamentary speeches	Institutional activity reports and advertising for raising awareness on gender	Scientific literature
	Descriptive and exploratory content analysis	Descriptive and exploratory content analysis	Descriptive content analysis
Methodology	Systematic search of the parliamentary database	Analysis based on institutional actions for raising awareness on gender	Systematic review of articles (published between 1998 and 2008) which analyse advertising in medical journals
	Analysis based on a retrospective study of the frequency of legislative initiatives and the prevalence of different arguments and positions in abortion debates	Institutional resources: (1) city councils, (2) provincial councils, (3) regional directorates for women, (4) other council departments promoting co-responsibility, with a regional scope of action	Databases: PUBMED, Medline, Scopus, Sociological Abstract, Eric and LILACS
		Advertising database: Infoadex (agency which studies the evolution of investment in advertising in Spain)	
Studies	Study I: ‘Abortion in Democratic Spain: The Parliamentary Political Agenda (1979–2004)’	Study II: ‘Public actions of gender awareness. Efforts of regional and local governments in advertising communication (1999–2007)’	Study III: ‘Quality of pharmaceutical advertising and gender bias in medical journals (1998–2008): a review of the scientific literature’
Periods of time	1979–2004	1999–2007	1998–2008

To apply a gender perspective, we used the level of gender observation proposed by S. Harding, applied in a previous health study (35), which states that: (1) gender is the basis of social norms and (2) gender is one of the organisers of the social structure (36).

### Study I. Abortion in democratic Spain: the parliamentary political agenda 1979–2004

Since Spain's transition to democracy in 1978, arguments for and against the legalisation of abortion and its coverage under public health services have taken place both inside and outside the Spanish Parliament (37, 38). We thought it would be useful to analyse Parliamentary debates and voting patterns to identify the positions of political parties and the agreements and disagreements within each party, as well as to examine the positions of male and female Members of Parliament. This will help to identify the key points of political debate and ways to encourage the promotion of abortion legislation that takes into account the needs of women. We also thought it would be useful to carry out political epidemiological research on the effects of decisions made by political institutions on people's health.

The hypothesis is that gender is the basis of institutional norms created during the Spanish democracy (39). Thus, greater participation of women in the abortion debate increases their ability to influence political decisions on reproductive health (37).

Our analysis is based on a retrospective study of the frequency of legislative initiatives on abortion in democratic Spain. We also carried out a descriptive content analysis of different arguments and positions in abortion debates, found through a systematic search of the parliamentary database between 1979 and 2004. The parliamentary speeches delivered by Members of the Spanish Parliament were analysed according to the speaker's sex and political affiliation.

### Study II. Public actions of gender awareness. The efforts of regional and local governments in advertising communication (1999–2007)

This study starts from the fact that gender inequalities in health in Spain are associated with the unequal distribution of family demands and the lack of active social policies that facilitate the equal distribution of unpaid housework (40, 41). The Law on the Reconciliation of Work and Family Life, enacted in 1999, aims to facilitate the solution to the problem (42). Amongst other things, it suggests carrying out institutional campaigns aimed at increasing adequate social solutions for maintaining, protecting, and promoting health by raising awareness about the need to share reproductive work (42, 43).

Feminist Media Studies argue that the gender system can be changed by intervening in the frequency and way that women are portrayed (44, 45). In accordance with this line of research, our objective was to examine the actions of communication aimed at raising gender awareness as indicators of institutional efforts to promote equality (1999–2007).

We analysed the actions implemented by public institutions in six Spanish provinces aimed at raising awareness of gender and encouraging shared responsibility as a means of promoting equality in the distribution of domestic tasks and care, thus tackling the role of gender as a determining factor in health inequities (46). Database: (1) City councils of the capitals of the 6 regions, (2) provincial councils, (3) regional directorates for women or similar institutions, (4) other council departments promoting co-responsibility, and (5) Infoadex Agency. The analytical framework considered the following dimensions: visibility, parity, mainstreaming, and empowerment.

### Study III. Quality of pharmaceutical advertising and gender bias in medical journals (1998–2008): a review of the scientific literature

Due to the increasing global and fragmented context in which they work, physicians are partially dependent on the flow of information conveyed through advertising, which acts as a socialising agent and transmits messages that contribute to the social construction of disease (47). Marketing strategies target the medical community and do not always offer neutral information in order to increase sales (48–50).

One of the marketing strategies employed to achieve greater impact is the incorporation into advertising of images that segment the consumer according to socio-demographic characteristics. Furthermore, the potential population base that could benefit from the medication is increased through the use of inappropriate frames that include non-risk groups, to whom the therapeutic indications do not apply. This phenomenon is known as disease mongering (51, 52).

The representation of both sexes in pharmaceutical advertisements is a point of interest in research on gender and health issues (46, 53, 54) given that, if such representations are inconsistent with reality, they may reinforce the perception that certain illnesses are associated with the most frequently portrayed sex. As an innovative research field focused on studying the differences between men and women and how such differences affect diseases and their diagnoses and treatments, gender-based medicine and evidence-based medicine share the hypothesis that there are inaccuracies in the production and dissemination of knowledge, as well as in medical practice, with regard to rigour, transparency, and subjective judgement (55–57).

The aim of this study was to determine whether gender bias has decreased and whether the quality of information in pharmaceutical advertising targeted at health professionals has improved over time. We carried out a descriptive review of the scientific literature available on pharmaceutical advertising between 1998 and 2008. The articles’ findings were considered according to the following quality criteria: 1) the number, validity, and accessibility of bibliographic references provided in pharmaceutical advertisements, and 2) the relationship between the sexes portrayed in the advertisements and the sex prevalence of the diseases treated by the drugs advertised.

## Main results and discussion

### Article I

We analysed a total of 229 legislative initiatives in which abortion was mentioned in the period between 1979 and 2004. A total of 215 parliamentarians (143 women, 72 men) intervened in abortion issues during this period. Despite the fact that women were a minority in all 8 Parliaments, they dominated the abortion debate and introduced most of the legislative initiatives (60%).

The inclusion of socio-economic grounds for legal abortion (64%) and making abortion on request legal in the first 12 weeks of pregnancy (60%) were the most frequent proposals for law reform, mostly based on pro-women's rights arguments. In contrast, male and female members of anti-abortion parties and most male members of other parties supported foetal rights significantly more often (*p*=0.001). Unsafe abortion and international agreements on women's rights as well as women's health received very little attention.

The debate was led by the Justice Commission rather than the Health or Social Affairs Commission, meaning that legal aspects prevailed over women's health issues. Female parliamentarians not only spoke more often, but they also advocated pro-choice reforms of current laws significantly more often than men (*p*=0.001). While female Members of Parliament belonging to left-wing parties led the political debate on abortion, they were not responsible for many decisions. This was probably due to the fact that most Members of Parliament were men and that, contrary to right-wing parties, male and female members of left-wing parties were not in agreement.

The social construction of unsafe abortion as a public health problem, promoted by Parliamentarians and non-Governmental women and media, may have influenced the enactment of the Spanish Sexual and Reproductive Health and Abortion Law in March 2010, which legalised abortion on request in the first 12 weeks of pregnancy. This law is being discussed again by the Conservative Party currently in Government.

### Article II

We analysed 5,697 educational and communication actions. Seventy-nine percentage of institutional initiatives aimed at promoting equality awareness were in the form of educational actions, while unconventional advertising accounted for 6%. We also identified 136 advertisements linked to the aim of the study.

The predominance of women as the target group of institutional gender awareness campaigns proves that the gender perspective still lacks the promotion of shared responsibilities between men and women.

When it comes to investment in mass media, Madrid was responsible for 56.17% of the total number of campaigns aimed at promoting gender awareness, followed by the Spanish regions with the highest gender-related development: Catalonia (19.62%) and the Basque Country (12.73%). However, public funding has not been used to promote gender equality. This is because the gender awareness actions carried out to date have prioritised women's training and employability. Differences between regions suggest that, in addition to developing a certain number of public policies, there is a need for institutions that act as ‘policy watchdogs’ to ensure the proper implementation and maintenance of national equality laws.

### Article III

The scientific literature review focused on 31 articles – published between 1998 and 2008 – which analyse advertising in medical journals from 1975 to 2005. Nine articles provided information on the sex of the people who appeared in the advertisements and the gender dimension used as categories of analysis (22, 23, 30, 31, 33, 36, 41, 43, 44). No improvement was observed when the quality criteria were examined from a gender perspective, because they all confirmed that there was a tendency to depict men in paid productive roles, while women appeared inside the home or in non-occupational social contexts. Advertisements for psychotropic and cardiovascular drugs over-represented women and men, respectively. In addition, we found that the number of references used to support pharmaceutical advertising claims increased from 1975 onwards but that 50% of these references were not valid.

Despite the social changes experienced by men and women since 1970s, medicine is still viewed as a gendered organisation with a male-dominated culture, which has had a powerful effect on gender imagery for women. This perspective has defined medicine as a cultural system with a tendency to reinforce gender identity based on the traditional gender roles of women in society (52). The accuracy of knowledge transfer through pharmaceutical advertising is essential in order to avoid gender bias in medical practice and to achieve quality drug prescriptions according to knowledge-based evidence (56).

Gender awareness actions carried out to date have prioritised women's training and employability, and public funding has not been used to promote gender equality or the development of public policies. Thus, the false assumptions internalised by both health workers and consumers connect the economic interests of pharmaceutical companies with the gender system. Advertising and gender feed off each other through the process of social construction that characterises them both. Insufficient financial investment and the lack of medium- to long-term communication plans in institutional gender awareness actions do not contribute to social change.

There is a need to strengthen the mainstreaming of public policies and to increase the gender sensitivity of equality policies. In the meantime, pharmaceutical companies are devoting large budgets to developing advertising and marketing strategies in order to increase sales and become one of the most important filters of medical knowledge. However, our studies have demonstrated the presence of gender bias in advertising (discrepancy between prevalence and representations by sex and consistency with the gender stereotype). This has resulted in an increase in the number of diagnoses and treatments, thereby increasing gender inequities and primarily affecting women's health.

## Conclusions

Each of the three articles draws their own conclusions. However, the global conclusions are as follows:

Gender sensitivity implies intervening in the social construction of reality and is a key factor for changing the established gender system. This becomes even more important when the lack of gender sensitivity impacts on women's health. Greater gender sensitivity could be achieved through greater participation of women in public policy and decision-making and in the control of information released by the media. This would empower women and increase their ability to establish the necessary measures for raising awareness and thus achieving social change.

According to the feminist standpoint epistemology, an increase in the number of women in Parliament (i.e. greater equality) would result in discussions that are closer to women's needs and the implementation of health policies aimed at solving women's specific problems. It could also increase the willingness to change, providing financial resources for carrying out institutional media campaigns to raise awareness of health problems derived from a lack of equality and introducing more equal social models.

This willingness to change could also be expressed through the creation of a binding code of ethics for the pharmaceutical industry, which would contribute to the elimination of gender biases in advertising. First, these biases are detrimental to women's health given that they reinforce the concept of gender as an organising principle of social structures, through which male and female activities in society are segregated according to their sex. Second, gender biases allow the industry to take advantage of the gender system in order to define a market niche in situations in which male and female stereotypes are redefined (motherhood, menopause, sexuality, or old age).

## References

[1] World Health Organization (2007). A conceptual framework for action on the social determinants of health.

[2] Dahlgren G, Whitehead M (1991). Policies and strategies to promote social equity in health.

[3] Doyal L (2001). Sex, gender, and health: the need for a new approach. BMJ.

[4] Krieger N (2003). Genders, sexes and health: what are the connections – and why does it matter?. J Epidemiol Community Health.

[5] Velasco S (2009). Recommendations for the practice of gender in health programs.

[6] Peiró R, Ramón N, Álvarez-Dardet C, Colomer C, Moya C, Borrell C (2004). Gender sensitivity in the formulation of Spanish health plans: what it could have been but wasn't. Gac Sanit.

[7] World Health Organization (2009). Women and health: today's evidence tomorrow's agenda.

[8] Annandale E, Hunt K (2000). Gender inequalities in health.

[9] Whitehead M (1990). The concepts and principles of equity and health.

[10] Marmot M, Friel S, Bell R, Houweling T, Taylor S (2008). Closing the gap in a generation: health equity through action on the social determinants of health. Lancet.

[11] Carrasco-Portiño M, Ruiz-Cantero MT, Gil-González D, Alvarez-Dardet C, Torrubiano-Dominguez J (2008). Gender development inequalities epidemiology in Spain (1990–2000). Rev Esp Salud Pública.

[12] Carrasco-Portiño M, Ruiz-Cantero MT, Fernández-Sáez J, Clemente-Gómez V, Roca V (2010). Geopolitical development inequalities in gender in Spain 1980–2005: a structural determinant of health. Rev Esp Salud Pública.

[13] Papí-Gálvez N, Cambronero-Saiz B, Frau-Linares MJ (2011). Gender Equality on institutional communication. Making aware in co-responsability.

[14] Artazcoz L, Borrell C, Benach J (2001). Gender inequalities in health among workers: the relation with family demands. J Epidemiol Community Health.

[15] Martín-Llaguno M (2007). Women in the advertising industry. Vertical segregation in commercial communication: glass ceiling and tacky floor. Zer.

[16] Peiró R, Vives C, Alvarez-Dardet C, Mas R, Borrell C, Artazcoz L (2007). Policy analysis from gender and health perspective. SEE Monograph: Research on gender and health.

[17] Papí-Gálvez N (2005). Conciliation of work and family life as a quality of life project from equality. RES.

[18] Artazcoz L, Artieda L, Borrell C, Cortés I, Benach J, García V (2004). Combining job and family demands and being healthy. What are the differences between men and women?. Eur J Public Health.

[19] Artarcoz L, García-Calvente MM, Esnaola S, Borrell C, Sánchez-Cruz JJ, Ramos JL, Cabasés JM, Villalbí JR, Aibar C (2002). Gender inequalities in health: the reconciliation of work and family life. Health Investment. Priorities in Public Health.

[20] Artazcoz L, Cortès I, Moncada S, Rolhlfs I, Borrell C (1999). Gender differences in the influence of housework on health. Gac Sanit.

[21] García-Calvente M, Mateo-Rodríguez I, Eguiguren A (2004). The system of informal caregiving as inequality. Gac Sanit.

[22] Messing K (2002). The work of women. Understanding to transform. Institute of Work, Environment and Health.

[23] Backhans M (2011). Gender policy and gender equality in a public health perspective.

[24] Braidotti R, Griffind G, Braidotti R (2002). Sex/gender terminology and its implications. Thinking differently. A reader in European women's studies.

[25] Berger P, Luckmann T (1966). The social construction of reality.

[26] McQuail D (1987). Mass communication theory: an introduction.

[27] Harding S (1993). The science question in feminism.

[28] Carrasco-Portiño M (2011). Gender perspective of human development as structural determinant of women's health: maternal mortality and intimate partner violence.

[29] Papí N (2008). Gender through wings. The case of women journalists in the Valencian Community.

[30] Rakow L, Wackwitz L (2004). Feminist communication theory: selections in context.

[31] Villaplana V (2008). Feminist identities, visual culture and narratives. Asparkia.

[32] Ramos M (2006). Women's history and feminist thought: a plural history to debate. Vasconia.

[33] Hartsock N (1998). The feminist standpoint revisited and other essays.

[34] Sánchez L, Reigada A, Leyva MJ, Olaizola R (2007). Revisiting communication from the feminist criticism. Introductory notes. Feminist Criticism and Communication.

[35] Ruiz-Cantero MT, Papí-Gálvez N, Carbrera-Ruiz V, Ruiz-Martínez A, Álvarez-Dardet C (2006). Gender systems and/in the Spanish National Health Interview Survey. Gac Sanit.

[36] Harding S (1991). Whose science? Whose knowledge? Thinking from women's lives.

[37] Cambronero-Saiz B, Ruiz-Cantero MT, Vives-Cases C, Carrasco-Portiño M (2007). Abortion in democratic Spain: the parliamentary political agenda 1979–2004. Reprod Health Matters.

[38] McBride Stetson D (2001). Abortion politics, women's movements, and the democratic state.

[39] Peiro R, Colomer C, Alvarez-Dardet C, Ashton JR (2001). Does the liberalisation of abortion laws increase the number of abortions? The case of Spain. Eur J Public Health.

[40] Papí-Gálvez N, Cambronero-Saiz B (2011). Public actions of gender awareness. The efforts of regional and local government in advertising communication (1999–2007). PLP.

[41] Larrañaga I, Arregui B, Arpal J (2004). Reproductive or domestic work. Gac Sanit.

[42] Law on reconciliation of work and family life (Law 39/1999), BOE 266 (1999/11/06), 38934-38942. Spain, Head of State (1999).

[43] Martinez E, Vizvaíno-Laorga J, Gavilán R (2008). Government advertising: an integrative element. The legal framework in Spain. Rev Lat Comun Soc.

[44] Mauro W (1994). The Social Effects of Media.

[45] Van Zoonen L (1994). Feminist media studies.

[46] Lorber J (1997). Gender and the social construction of illness.

[47] Cambronero Saiz B, Ruiz Cantero MT, Papí-Gálvez N (2012). Quality of pharmaceutical advertising and gender bias in medical journals (1998–2008): a review of the scientific literature. Gac Sanit.

[48] Moynihan R, Heath I, Henry D (2002). Selling sickness: the pharmaceutical industry and disease mongering. BMJ.

[49] Grier S, Bryant C (2005). Social marketing in public health. Annu Rev Public Health.

[50] FughBerman A, Alladin K, Chow J (2006). Advertising in medical journals: should current practices change?. PLoS Med.

[51] Doran E, Henry D (2008). Disease mongering: expanding the boundaries of treatable disease. Intern Med J.

[52] Ruiz-Cantero MT, Cambronero-Saiz B (2011). Health metamorphosis: disease mongering and communication strategies. Gac Sanit.

[53] Riska E, Texler Segal M, Demos V (2003). Gendering the medicalization thesis. Gender perspectives on health and medicine. Adv Gend Res.

[54] Hawkins J (1993). Women in advertisements in medical journals. Sex Roles.

[55] Manderbacka K (2005). Exploring gender and socioeconomic differences in treatment of coronary heart disease. Eur J Public Health.

[56] Ruiz-Cantero MT, Vives-Cases C, Artazcoz L (2007). A framework to analyse gender bias in epidemiological research. J Epidemiol Community Health.

[57] Johnson JL, Greaves L, Repta R Better science with sex and gender: facilitating the use of a sex and gender-based analysis in health research. Int J Equity Health.

